# When Gastroenteritis Struck the Brain: Post-infectious Parkinsonism Suggestive of Neuropsychiatric Systemic Lupus Erythematosus in a Patient With Aqueductal Stenosis

**DOI:** 10.7759/cureus.111967

**Published:** 2026-07-02

**Authors:** Mannan Mangal, Prakhar Gupta, Charitha M S

**Affiliations:** 1 Internal Medicine, Mysore Medical College and Research Institute, Mysore, IND; 2 Internal Medicine, Jagadguru Sri Shivarathreeshwara (JSS) Medical College and Hospital, Mysore, IND

**Keywords:** gut dysbiosis, hydrocephalus, mutism, neuropsychiatric lupus, parkinsonism, periventricular hyperintensities, square wave jerks, systemic lupus erythematosus

## Abstract

Parkinsonism is a rare neuropsychiatric manifestation of systemic lupus erythematosus (SLE), the pathogenesis of which has been proposed to involve immune-mediated vasculopathy. Gastrointestinal infections may exacerbate autoimmune responses, triggering disease flares. We present a 26-year-old woman who developed acute parkinsonism with mutism, oromandibular dystonia, and horizontal square wave jerks following a diarrheal illness. Neurological examination revealed cogwheel rigidity and dysphagia. Brain MRI showed periventricular hyperintensities, ventricular dilation, and aqueductal stenosis without basal ganglia infarcts or lesions. Antinuclear antibody was positive (1:100) with anti-SS-A and anti-SS-B antibodies, while complement levels remained within normal limits. Treatment with pulsed methylprednisolone, rituximab, hydroxychloroquine, and levodopa-carbidopa resulted in marked neurological improvement. The patient did not fulfill the 2019 European League Against Rheumatism/American College of Rheumatology classification criteria for SLE; however, the clinical features and robust therapeutic response were consistent with a presumptive diagnosis of neuropsychiatric SLE presenting as parkinsonism. This case underscores the importance of considering SLE in young patients with atypical parkinsonism, particularly following a gastrointestinal illness. It highlights that complement levels may not reliably reflect disease activity in all patients.

## Introduction

PD typically results from the progressive loss of dopaminergic neurons in the substantia nigra, manifesting as bradykinesia, rigidity, tremors, and postural instability. Secondary parkinsonism, though rare, can occur in autoimmune diseases such as systemic lupus erythematosus (SLE), a condition known for its wide-ranging neuropsychiatric manifestations [1]. Neuropsychiatric SLE (NPSLE) refers to the spectrum of central and peripheral nervous system and psychiatric syndromes attributed to SLE, encompassing the 19 syndromes recognized by the American College of Rheumatology (ACR), ranging from cerebrovascular disease and seizures to mood disorders and peripheral neuropathy; movement disorders, including parkinsonism, represent an uncommon subset within this spectrum, and the underlying mechanisms remain poorly understood. This case report examines a challenging clinical sequence in which a gut infection appears to have triggered a presumptive autoimmune flare, ultimately presenting as a complex form of parkinsonism with mutism and abnormal eye movements. We discuss how this cascade may have contributed to the observed periventricular hyperintensities, ventricular dilation, and aqueductal stenosis, alongside paradoxically normal complement levels, and contrast these findings with previously documented cases.

## Case presentation

A 26-year-old woman presented with a seven-day history of slurred speech that had progressively worsened to complete mutism. Indirect laryngoscopy demonstrated adduction of the vocal cords with no intrinsic laryngeal pathology. She had a concurrent seven-day history of tremors involving the hands and feet that subsequently spread to all limbs, characterized by rhythmic flexion-extension movements that resolved with activity and were absent during sleep, accompanied by arrhythmic jaw and tongue movements. Informants reported intermittent episodes of limb stiffening and progressive motor slowing, eventually resulting in bedridden status due to impaired ambulation. On examination, she exhibited cogwheel rigidity and hypertonia in all limbs, a masked facies with reduced blink frequency, horizontal square wave jerks (SWJ) bilaterally, oromandibular dystonia, and progressive dysphagia, more pronounced for liquids than solids, with associated drooling. Sustained cervical extension was noted, causing neck and back pain, for which baclofen was administered.

Three weeks before the onset of these neurological symptoms, the patient experienced a 10-day illness comprising continuous high-grade fever with chills and rigors, three to five episodes of non-projectile vomiting, and four to five episodes of watery diarrhea, treated with conventional remedies. She had experienced similar episodes of diarrhea over the preceding two years. There was no family history of parkinsonism or similar neurological illness. During hospitalization, a low-grade fever persisted. General physical examination was unremarkable, with no scarring alopecia, skin lesions, oral ulcerations, arthralgia, or chest pain. No cerebellar or meningeal signs were elicited.

**Figure 1:**
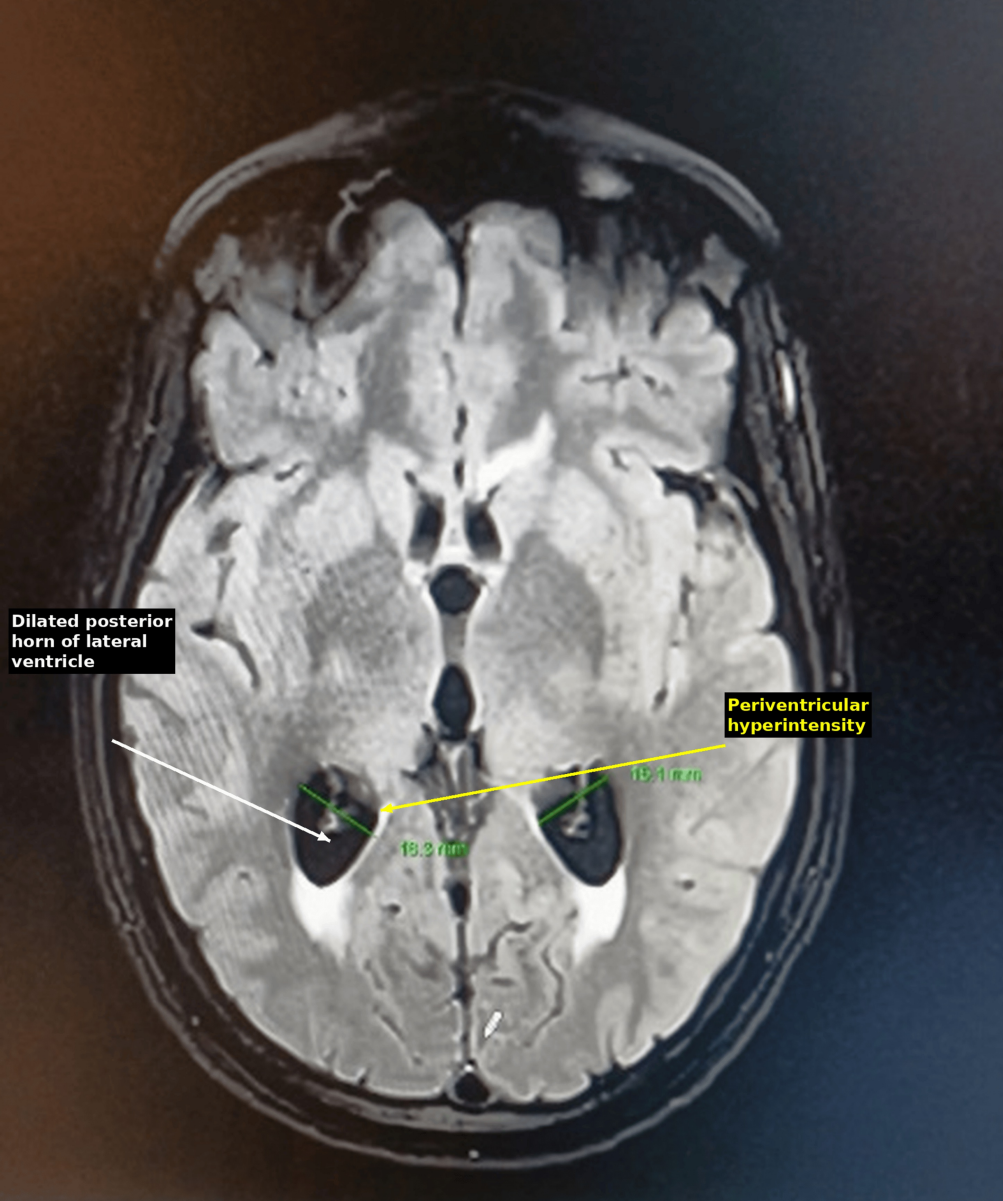
Axial FLAIR-weighted MRI of the brain demonstrating dilation of the posterior horn of the lateral ventricle (white arrow) with associated periventricular hyperintensity (yellow arrow), suggestive of periventricular flaring. No microinfarcts are identified in the cerebral parenchyma, basal ganglia, or brainstem MRI: magnetic resonance imaging, FLAIR: fluid-attenuated inversion recovery

Laboratory investigations revealed leukopenia, elevated erythrocyte sedimentation rate (ESR), and a positive direct Coombs test. ANA testing showed a speckled nuclear pattern at a titer of 1:100. Anti-SS-A and anti-SS-B antibodies were positive. Anti-double-stranded DNA, anti-Smith, anticardiolipin antibody, lupus anticoagulant, and anti-beta-2-glycoprotein-1 antibodies were negative. Serum C3 and C4 complement levels were normal on repeated testing. Blood lipopolysaccharide levels, measured using a commercial ELISA-based assay, were elevated; such assays are generally validated for research use, and reference ranges are not well standardized across platforms, which limits the clinical interpretability of this finding in isolation. T1-, T2-, and fluid-attenuated inversion recovery (FLAIR)-weighted MRI of the brain showed no microinfarcts in the cerebral parenchyma, basal ganglia, or brainstem; however, bilateral lateral ventricular dilation with periventricular FLAIR hyperintensities and third ventricular dilation were noted (Figure 1).

**Figure 2:**
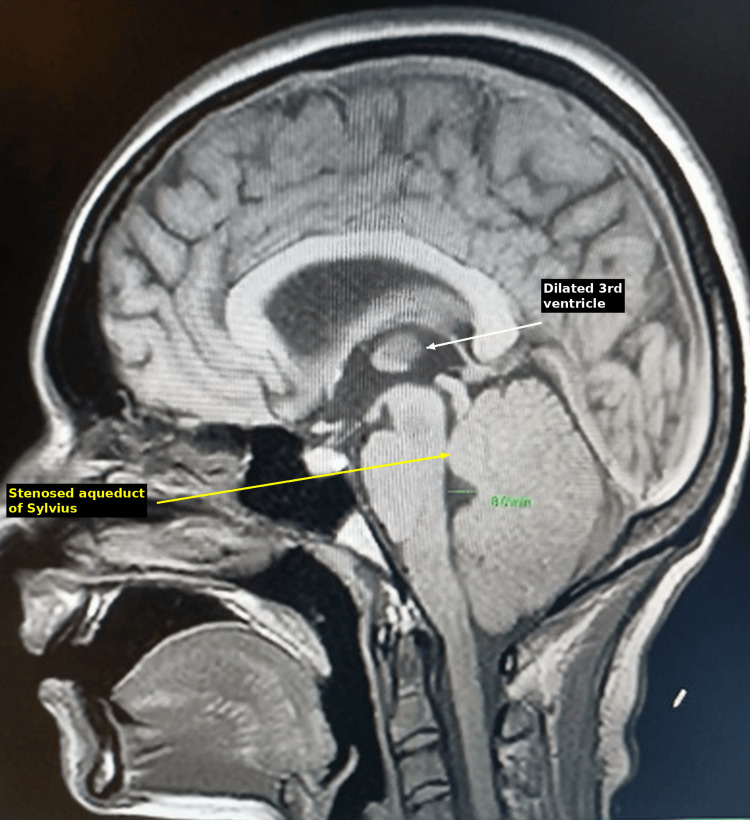
Sagittal T1-weighted MRI of the brain revealing stenosis of the aqueduct of Sylvius (yellow arrow), with associated dilation of the third ventricle (white arrow), suggestive of obstructive hydrocephalus MRI: magnetic resonance imaging

Cerebrospinal fluid (CSF) analysis showed a total of 2 nucleated cells/µL (occasional lymphocytes), with normal adenosine deaminase (ADA), lactate dehydrogenase (LDH), glucose, and protein levels; cultures and acid-fast bacilli stains were negative. These findings showed no pleocytosis or elevated protein and supported the exclusion of infectious etiology in the clinical context. Intracranial pressure was within normal limits. Single-photon emission computed tomography (SPECT) and 18F-fluorodeoxyglucose positron emission tomography (18F-FDG PET) could not be performed due to financial constraints. Sagittal MRI additionally revealed stenosis of the aqueduct of Sylvius with obstructive hydrocephalus (Figure 2).

Infectious meningoencephalitis was reasonably excluded by the normal CSF parameters and negative cultures described above, and the absence of any history of dopamine-blocking or other offending medications excluded drug-induced parkinsonism. Serum ceruloplasmin, 24-hour urinary copper, and slit-lamp examination for Kayser-Fleischer rings were not performed; therefore, Wilson disease was not formally excluded. Autoimmune encephalitis was not specifically investigated with a paraneoplastic or neuronal surface antibody panel, and mitochondrial disease was not pursued with genetic or biochemical workup; the acute-subacute, monophasic course with sustained remission made these less clinically likely, but they cannot be definitively excluded based on the investigations performed.

The patient was admitted to the intensive care unit for two weeks. Treatment was initiated with trihexyphenidyl 2 mg for tremors and levodopa-carbidopa 200/50 mg for parkinsonism. Given the severity and rapidly progressive nature of the presentation, together with an unrevealing infectious workup, empiric immunosuppression was pursued: hydroxychloroquine 200 mg for presumptive SLE; intravenous methylprednisolone pulse therapy daily for four days, followed by rituximab 375 mg/m² intravenously; and intravenous dexamethasone 8 mg for one week, followed by oral prednisolone 40 mg. The 2023 European Alliance of Associations for Rheumatology (EULAR) recommendations for NPSLE management generally favor glucocorticoids and conventional immunosuppressive agents as initial therapy, reserving rituximab for refractory disease; rituximab was used early in this patient, ahead of a trial of conventional immunosuppression, given the severity and rapid progression of the presentation, representing a deviation from this stepwise approach rather than direct adherence to it.

Neurosurgical evaluation for the obstructive hydrocephalus noted on imaging was not pursued at this stage, given that intracranial pressure was normal and the patient lacked clinical features suggestive of decompensated hydrocephalus; however, longer-term follow-up imaging would be warranted to monitor for progression. The patient experienced substantial clinical improvement, with resolution of mutism, rigidity, and dystonia, and a return to independent activity, following concurrent administration of dopaminergic and immunosuppressive therapy. No validated outcome scale, such as the Unified Parkinson's Disease Rating Scale or modified Rankin Scale, was administered at follow-up; because these agents were not given sequentially, the relative contribution of each and the possibility of spontaneous improvement cannot be determined from this case alone. During subsequent outpatient consultations, she reported no recurrence of neurological or systemic symptoms.

Although she did not fulfill the 2019 EULAR/ACR classification criteria for SLE, the combination of a high ANA titer (1:100), constitutional fever, leukopenia, positive anti-SS-A/anti-SS-B antibodies, and clinical improvement with combined therapy was consistent with a presumptive diagnosis of NPSLE presenting as parkinsonism; an unclassified autoimmune syndrome or undifferentiated connective tissue disease with neurological involvement remains an equally plausible label for this presentation, and we use NPSLE here as a working diagnosis rather than a confirmed nosological entity. We did not intend for treatment response to serve as confirmation of this diagnosis, and we recognize that improvement following combined immunosuppressive and dopaminergic therapy is consistent with, but does not establish, an underlying autoimmune cause.

## Discussion

The first documented case of parkinsonism secondary to SLE was reported in 1930 by Seminario and Pesano [2]. Since then, approximately 40 such cases have been documented globally, predominantly in young to middle-aged women, consistent with the broader demographic distribution of SLE itself; reported imaging findings have ranged from normal studies to basal ganglia lesions, periventricular changes, and, less commonly, hydrocephalus, and reported treatment responses have varied widely, with most cases showing at least partial improvement with glucocorticoids and immunosuppressive therapy. However, levodopa responsiveness has been inconsistent across reports. A summary of representative previously reported cases, alongside the present case, is provided in Table 1.

**Table 1:** Reported cases of SLE-associated parkinsonism compared with the present case The early reports [2,9] predated the availability of standardized neuroimaging and CSF analysis, accounting for the data gaps in this entry; these gaps are noted rather than inferred or estimated. SLE: systemic lupus erythematosus, NPSLE: neuropsychiatric systemic lupus erythematosus, EULAR: European Alliance of Associations for Rheumatology, ACR: American College of Rheumatology, MRI: magnetic resonance imaging, CSF: cerebrospinal fluid, FLAIR: fluid-attenuated inversion recovery, ADA: adenosine deaminase, LDH: lactate dehydrogenase, AFB: acid-fast bacilli, SWJ: square wave jerk

Study	Age/sex	Lupus context	Parkinsonian phenotype	Associated neurologic features	MRI/neuroimaging findings	CSF findings	Treatment	Clinical outcome
Seminario and Pesano, 1930 [2]	Not reported	First reported lupus-associated parkinsonism case	Parkinsonism in the setting of acute lupus	Not reported	Not reported	Not reported	Not reported	Not reported
Khubchandani et al., 2007 [9]	Juvenile/not fully specified in title	Juvenile SLE	Parkinsonism in a young lupus patient	Unusual neurologic manifestations; details of focal movement disorder not provided in the title	Not reported	Not reported	Not reported	Not reported
Wantaneeyawong et al., 2022 [19]	Multiple cases in review	SLE-associated parkinsonism	Acute or subacute parkinsonism across reported cases	Variable across cases; reports included speech impairment, bulbar involvement, dystonia, pyramidal signs, or other neuropsychiatric features depending on the case	Approximately half of reported cases had no basal ganglia lesion on MRI; when present, imaging abnormalities were heterogeneous and did not always match the clinical syndrome	Variable / inconsistently reported across cases	Variable; most cases improved with corticosteroids and/or immunosuppressive therapy	Variable; many cases improved, though severity and speed of recovery differed between reports
Present case	26-year-old woman	Presumptive NPSLE; did not fulfill 2019 EULAR/ACR SLE classification criteria	Severe akinetic-rigid parkinsonism with cogwheel rigidity, masked facies, bradykinesia, tremor, hypertonia, dysphagia, and reduced blink rate	Progressive mutism, arrhythmic tongue and jaw movements, oromandibular dystonia, horizontal SWJ, drooling, and sustained cervical extension causing pain	Bilateral lateral ventricular dilation, third ventricular dilation, periventricular FLAIR hyperintensities, and aqueductal stenosis with obstructive hydrocephalus; no basal ganglia infarct or focal parenchymal lesion identified	No pleocytosis; protein and glucose normal; ADA, LDH, Gram stain, cultures, and AFB studies negative; opening pressure normal	Trihexyphenidyl and levodopa-carbidopa, followed by pulse methylprednisolone, rituximab, hydroxychloroquine, dexamethasone, and oral prednisolone	Marked neurological improvement, including recovery from mutism and substantial improvement in rigidity, tremor, dysphagia, and overall function; no recurrence reported at follow-up

Among these, mutism was identified in 8.1% of SLE-associated parkinsonism cases [3], compared to 89% of idiopathic PD cases reporting speech disorders [4]. This lower frequency in SLE suggests a distinct pathological mechanism affecting the nigrostriatal pathway's regulation of speech motor function. Phonatory dysfunction in PD is typically linked to laryngeal muscle rigidity due to impaired basal ganglia control [4]. In contrast, articulatory dysfunction stems from extrapyramidal rigidity of facial and oral musculature [5]. The arrhythmic tongue and jaw movements observed prior to the onset of mutism in this patient point toward articulatory dysfunction as a contributing factor, which improved rapidly following levodopa therapy. The patient's progressive dysphagia and oromandibular dystonia likely compounded this picture, and it is difficult to fully separate the contribution of parkinsonian articulatory dysfunction from that of dysphagia-related and dystonic mechanisms in producing the mutism observed.

Horizontal SWJ are frequently observed in Parkinson-plus syndromes such as multiple system atrophy but occur in only approximately 20% of patients with idiopathic PD [6], making them even rarer in SLE-associated parkinsonism. In PD, loss of dopaminergic neurons in the substantia nigra pars compacta (SNpc) disrupts normal basal ganglia function, reducing inhibition of the globus pallidus internus and substantia nigra pars reticulata, thereby inducing excessive inhibition of the superior colliculus (SC), a key structure in saccadic eye movement control [7]. This impairs regulation of the SC's inhibitory signals to the frontal eye fields (FEF), and the resultant increase in pre-saccadic activity in the FEF has been hypothesized to cause involuntary saccades such as SWJ [8]. This pathway illustrates a plausible route by which SNpc involvement, occurring in the context of this patient's atypical parkinsonian syndrome, could produce SWJ as an unusual clinical finding. However, direct evidence of SNpc involvement was not obtained in this case.

Several theories have been proposed to explain the pathophysiology of SLE-associated parkinsonism, including direct immune-mediated mechanisms and indirect effects such as vasculopathy, coagulopathy, and bleeding disturbances. The most widely discussed explanation involves vasculopathy affecting the thalamostriate arteries, which are end arteries supplying the basal ganglia and are therefore particularly vulnerable to ischemia [9]. Histopathological confirmation was not available in this case; however, immune complex deposition and complement activation are thought to trigger endothelial proliferation and inflammation, thereby narrowing small-vessel lumens and reducing blood flow. These changes may be further compounded during acute infections by cytokine surges, including lipopolysaccharides, interleukin-1, and tumor necrosis factor-alpha (TNF-α), which enhance endothelial adhesiveness and promote platelet aggregation, further compromising perfusion [10]. The temporal relationship between this most recent illness and symptom onset is suggestive of a triggering or exacerbating role; however, the patient's two-year history of recurrent diarrheal episodes raises the possibility of a chronic underlying gastrointestinal or low-grade systemic inflammatory process that may have predisposed her to immune dysregulation over a longer period, with the final illness representing an acute exacerbation rather than a sole and sufficient trigger, culminating in central nervous system (CNS) involvement [11]. No causal biomarker, such as stool pathogen identification or paired antibody titers, was obtained to substantiate a post-infectious trigger beyond this temporal sequence, and temporal coincidence cannot be excluded.

Gut dysbiosis has been increasingly implicated in the pathogenesis of SLE. A gut microbiota study in Chinese SLE patients revealed enrichment of pathogenic genera, including *Rhodococcus*, *Klebsiella*, and *Bacteroides*, and a reciprocal depletion of *Lactobacillus* and *Firmicutes* [12]. Notably, *Lactobacillus* exerts anti-inflammatory effects in SLE by reducing pro-inflammatory markers, including those of innate lymphoid cell type 3 and T helper 17 cells, while stimulating interleukin-10 production [13]. Firmicutes demonstrate a negative correlation with SLE Disease Activity Index scores, suggesting a protective role [14]. Dysbiosis resulting from an acute enteric infection can compromise gut barrier integrity, increasing intestinal permeability and allowing lipopolysaccharides and other bacterial products to enter the systemic circulation, triggering a pro-inflammatory cascade that may precipitate or exacerbate autoimmune disease [15]. Contributing mechanisms include molecular mimicry, epitope spreading, bystander activation, superantigens, and autoreactive cell expansion [16]. This mechanistic narrative draws on prior published cohort data rather than on direct testing in this patient; no stool microbiome analysis, gut permeability markers, or longitudinal cytokine profiling were obtained in this case, and the elevated serum lipopolysaccharide level, while consistent with increased systemic exposure to bacterial products, does not by itself localize the origin of that exposure to the gut or confirm a dysbiosis-driven mechanism. The cited microbiome studies were conducted in Chinese SLE cohorts, and gut microbiome composition varies substantially by geography, diet, and population; the applicability of these findings to this Indian patient is not established and should be regarded as a further limitation of the proposed mechanistic narrative. The gut dysbiosis hypothesis should therefore be regarded as a plausible explanation drawn from the broader literature rather than a finding demonstrated in this patient.

Despite clinical features consistent with presumptive CNS involvement in the context of SLE, this patient’s serum C3 and C4 levels remained within the normal range on repeated testing. This is not without precedent; similar cases of SLE with presumed CNS vasculopathy and normal complement levels have been described [17], and several explanations have been put forward. These include genetic variation in baseline complement levels, the nature of C3 and C4 as acute-phase reactants whose synthesis may increase during inflammation to offset ongoing consumption by immune complexes, and the possibility that serum complement levels simply do not capture tissue-specific activation, particularly in disease that is predominantly localized [18]. More sensitive markers of complement activation, such as CH50, C1q, or split products including C3a, C3d, and C5a, were not assessed in this patient and may have provided more informative evidence of complement consumption than static C3 and C4 levels alone; in their absence, we are unable to confirm which mechanism best explains this patient's normal complement levels. Of the three possibilities discussed, we consider acute-phase upregulation of C3 and C4 offsetting ongoing consumption the most plausible explanation in this case, given the patient's acute presentation with markers of systemic inflammation, including fever, leukopenia, and an elevated ESR, occurring alongside normal complement levels; however, this remains an inference rather than a confirmed mechanism, and genetic variation in baseline complement levels or tissue-localized activation cannot be excluded.

MRI in this case revealed no microinfarcts or lesions in the basal ganglia, consistent with findings in nearly half of similar reported cases [19]. The principal imaging findings were ventricular dilation, aqueductal stenosis, and periventricular signal abnormalities. The parkinsonian phenotype observed in this patient, including cogwheel rigidity, masked facies, and response to levodopa-carbidopa, is consistent with nigrostriatal dysfunction on clinical and physiological grounds; this inference is not supported by direct structural or functional imaging of the substantia nigra or striatum, and a contribution from brainstem, frontal-subcortical, or hydrocephalus-mediated pathways cannot be excluded. This highlights the potential value of advanced modalities such as diffusion-weighted imaging and functional MRI for detecting subtle structural changes and SPECT and 18F-FDG PET for identifying early functional abnormalities, such as impaired cerebral blood flow or altered glucose metabolism, that may accompany SLE-related neurological disease, even when conventional MRI appears relatively unremarkable [20]. Neither SPECT nor 18F-FDG PET could be obtained in this patient due to financial constraints, and their absence limits our ability to substantiate a functional nigrostriatal or basal ganglia disturbance beyond the clinical phenotype and treatment response described.

The periventricular hyperintensities and ventricular dilation observed in this case may reflect a combination of periventricular fluid accumulation, demyelination, and reactive gliosis arising from endothelial dysfunction, potentially mediated by the presumed autoimmune process. Trans-ependymal CSF leakage secondary to cytokine-driven endothelial injury is one proposed mechanism. The normal appearance of the fourth ventricle on MRI is consistent with obstructive hydrocephalus at the level of the aqueduct of Sylvius; however, the underlying cause of the aqueductal narrowing cannot be determined from the imaging studies alone. Periaqueductal edema secondary to endothelial injury and perivascular fluid leakage is a plausible but unconfirmed explanation; no empirical evidence in this patient, such as cine MRI of CSF flow or follow-up imaging, distinguishes this possibility from a congenital or chronic structural aqueductal stenosis unrelated to the presumed autoimmune process. The two cannot be differentiated based on a single MRI study. A similar pattern has been described in prior reports of post-inflammatory CNS involvement in autoimmune disease, and the finding of normal intracranial pressure in this patient is consistent with a chronic partial obstruction to which the brain has partially adapted over time.

Given the presence of aqueductal stenosis and obstructive hydrocephalus on imaging, hydrocephalic parkinsonism warrants consideration as an alternative or contributing explanation for this patient's movement disorder. Hydrocephalic parkinsonism, most often described in the context of normal pressure hydrocephalus (NPH), classically presents with a wide-based, magnetic gait, postural instability, and a triad of gait disturbance, cognitive decline, and urinary incontinence. None of these features were prominent in our patient. Furthermore, drooling and resting tremor, both present in this case, are atypical for NPH-related parkinsonism, and NPH-related parkinsonism characteristically shows a poor or absent response to levodopa. In contrast, our patient demonstrated marked improvement with levodopa-carbidopa and, notably, with immunosuppressive therapy, an intervention that would not be expected to influence a purely structural, obstructive process. Intracranial pressure remained normal throughout, and the patient lacked the gait and cognitive features that typically prompt evaluation for CSF diversion. Taken together, these features make a purely hydrocephalus-driven process less likely as the sole explanation and suggest that an autoimmune process is an important contributor to this patient's movement disorder, alongside aqueductal narrowing and obstructive hydrocephalus, rather than treating either as the established sole cause. This inference is clinical, drawn from the treatment response and the absence of features typical of hydrocephalic parkinsonism, and is separate from the question of what structurally caused the aqueductal narrowing itself, which remains undetermined. Obstructive hydrocephalus cannot be excluded as a contributing factor to the overall clinical picture, but we do not infer that the autoimmune process is responsible for the aqueductal narrowing; this remains one plausible, unconfirmed possibility among others, including a congenital or chronic structural narrowing unrelated to autoimmune disease.

## Conclusions

This case draws attention to an uncommon and diagnostically challenging presentation of presumptive NPSLE, manifesting as parkinsonism with mutism, oromandibular dystonia, and horizontal SWJ. A preceding gut infection may have contributed to immune dysregulation and CNS involvement through dysbiosis-mediated mechanisms, though the precise pathophysiology remains speculative. The principal MRI findings were ventricular dilation, aqueductal stenosis, and periventricular signal abnormalities; no basal ganglia lesions were identified, and the etiology of the aqueductal narrowing could not be established from imaging alone. Normal complement levels in this case serve as a reminder that serological markers do not always reflect the extent of end-organ involvement and that clinical judgment remains indispensable. Finally, this case suggests that a compelling clinical picture and a meaningful response to combined therapy can justify treating NPSLE as a working diagnosis even when formal classification criteria are not met, provided this label is used to guide further investigation and, where appropriate, empiric treatment, rather than to establish a final diagnosis. To avoid overdiagnosis in similar future cases, we suggest that such presentations be explicitly documented as presumptive and that alternative diagnoses be revisited if the expected treatment response does not occur. We recommend that formal reclassification be pursued if and when the patient subsequently meets established classification criteria, rather than relying on treatment response alone to confirm the diagnosis retrospectively.
